# Development of a Lab-on-a-Disk Platform with Digital Imaging for Identification and Counting of Parasite Eggs in Human and Animal Stool

**DOI:** 10.3390/mi10120852

**Published:** 2019-12-05

**Authors:** Sertan Sukas, Bieke Van Dorst, Agata Kryj, Ole Lagatie, Wim De Malsche, Lieven J. Stuyver

**Affiliations:** 1 µFlow group, Department of Bioengineering Sciences, Vrije Universiteit Brussel, Pleinlaan 2, 1050 Brussel, Belgium; sertansukas@gmail.com (S.S.); Agata.Kryj@vub.be (A.K.); 2 Janssen Global Public Health, Turnhoutseweg 30, 2340 Beerse, Belgium; bieke_vandorst@hotmail.com (B.V.D.); OLAGATIE@its.jnj.com (O.L.); lstuyver@its.jnj.com (L.J.S.)

**Keywords:** particle separation, parasite egg identification and quantification, diagnostic microfluidic device

## Abstract

We present a lab-on-a-disk technology for fast identification and quantification of parasite eggs in stool. We introduce a separation and packing method of eggs contained in 1 g of stool, allowing for removal of commonly present solid particles, fat droplets and air bubbles. The separation is based on a combined gravitational and centrifugal flotation, with the eggs guided to a packed monolayer, enabling quantitation and identification of subtypes of the eggs present in a single field of view (FOV). The prototype was tested with stool samples from pigs and humans infected with intestinal parasites (soil-transmitted helminths eggs). The quality of the images created by this platform was appropriate for identification and quantification of egg types present in the sample.

## 1. Introduction

According to World Health Organization (WHO), more than 1.5 billion people, or 24% of the world’s population, are infected with soil-transmitted helminths (STH) worldwide [1]. The prevalence of STH within affected populations has been traditionally determined with microscopic tools, which identify and quantify parasite eggs or larvae in stool samples. The number of eggs in stool is a proxy measure for the determination of the infection levels. Depending on the type of the parasite, the total number can go well below 100 eggs per gram (EPG) for the light infection of *Schistosoma mansoni*, but also even above 50,000 EPG for heavy infections of *Ascaris lumbricoides* [2]. Besides quantification, accurate identification of the type of the egg is of vital importance in selecting the correct treatment approach.

The most commonly used method for quantification and identification of parasite eggs in stool is the Kato–Katz (KK) method, which is based on manual counting of the parasite eggs in a sieved stool sample using light microscopy [3]. KK is simple and inexpensive; however, the examination of the KK slides requires a significant time investment and a skilled parasitologist. Furthermore, the method lacks accuracy, resulting in underestimation of the prevalence in populations with low intensity infections after repeated rounds of mass drug administration (MDA) [4,5,6,7]. Even though, KK is a method recommended for planning, monitoring and the evaluation phase of the STH elimination plan, the only method with the ability to confirm discontinuation of the STH program is qPCR so far. It is however a time-consuming technique, requiring expensive equipment. That is why there is a need to develop a tool, which is less expensive but as sensitive as qPCR [8].

To overcome some of the limitations of KK, flotation-based technologies have been developed. Although information is limited for describing the composition of stool, it is well known that the density of the parasite eggs is lower than that of the majority of the stool particles. Therefore, transferring stool in a dedicated solution with a density slightly higher than the density of the eggs results in flotation of the eggs whereas most of the particles sediment due to their higher density. This is a very simple and efficient mechanism for enrichment of the eggs.

The Cornell Wisconsin centrifugal flotation method was first described in 1981 [9] and was updated in 1982 [10]. The principle of this method is that the flotation of eggs in salt solutions is enabled by centrifugation. Indeed, after centrifugation in a swinging bucket rotor, the eggs were collected on the top of the centrifugal tube meniscus, and subsequently transferred onto a cover slip and counted by light microscopy. The Cornell Wisconsin method has been compared to several flotation-based devices that were developed so far and was systematically categorized as being less sensitive than its successor technologies, namely McMaster, FLOTAC and FECPAK as the most successful implementations [11]. However, the transfer procedure of the eggs from the centrifugation tube to the microscope slide leads to an error-prone procedure, and a low sensitivity compared to the other flotation methods.

In McMaster, the sample is added to a flotation solution and placed under a slide with two gridded chambers. Eggs float towards the surface and the ones within the gridded area of the chamber are counted using a microscope [12].

FLOTAC technology is based on centrifugal flotation of a fecal sample suspension and subsequent translation of the apical portion of the floating suspension [13]. The FLOTAC apparatus is a cylindrical device consisting of two 5 mL flotation chambers. The system allows analysis of fecal material up to 1 g, compared to 42 mg in KK. The developers claim that FLOTAC techniques provide the highest sensitivity and accuracy available on the market compared to the methods based on physical separation of the parasite eggs [13]. The main limitations of the FLOTAC technique are the complexity of the application and the requirement for centrifugation of the sample using a large swinging bucket centrifuge, which is often not available in laboratories in developing countries. To overcome this bottleneck, a simplified version of the primary technology has been developed: mini-FLOTAC. One of the main advantages of this new method is the elimination of the centrifugation requirement, allowing easier transfer and simpler application [14]. However, omitting the centrifugation step and relying only on the natural flotation of the eggs by Earth’s gravity by shrinking the chamber volumes, resulted in a decrease in sensitivity [15].

The results of all the methods described above are read by traditional microscopy and are reported as writings on paper. For real-time monitoring of the prevalence and the intensity of intestinal STH infections during surveillance programs, digitalization of the results is essential. This would allow for instant data transfer to the cloud server and to create an archive for reinspection in case of doubt. FECPAK is currently the only method that provides the digitalization of the results of egg counting. In FECPAK, a special tube with a central pillar is filled with a stool sample dissolved in flotation solution, allowing the parasite eggs to accumulate into a single viewing area within a fluid meniscus. An image of the fecal sample is then captured and stored in a computer, through which it can also be uploaded to the cloud. Setting up the FECPAK test does not require special laboratory equipment or technical skills and the storage of digital images allows data processing at a later time [16]. The advantage of FECPAK technology is the delivery of the image for the future application of automated identification and counting of the eggs. However, FECPAK delivers lower sensitivities and egg recovery rates compared to KK [17]. Comparison of the major methods for copromicroscopic diagnosis of parasites can be found in Table 1.

A group of key opinion leaders represented the diagnostic users by describing possible scenarios faced by STH programs, creating a series of problem statements and decisions that each can be addressed by a hypothetical diagnostic. Each solution is detailed in a target product profile (TPP) as a list of technical characteristics, such as type of measurement and implementation requirements. TPPs are intended to provide the community with a pathway for research, development, evaluation, and implementation of diagnostic tools designed for STH programs. As described in the TPP for STH use case 1 technology [18], the ideal stool-based test is an integrated technology, which contains at least the following aspects:

1. Start from a gram-amount of stool to have a representative aliquot;
2. Sample-in result-out test principle;
3. Fully automated;
4. Digital output for test results, either by pictures of the ROI (region of interest), or a table;
5. Quality controlled procedure containing a positive control for sample manipulation, egg collection, and reproducibility in counting of parasitic eggs;
6. Data upload to a monitoring system; and
7. Re-usable assay components or environmentally friendly single-use disposables.

The TPP is hence standing for a complete and integrated point-of-care technology that should be addressed in multiple research and development steps.

We present a lab-on-a-disk (LOD) technology that has the potential to comply with the TPP, based on centrifugation and flotation of eggs to isolate and collect the eggs within an imaging zone, of which a single digital image is captured at the end of the process. Lab-on-a-disk (LOD) based applications are developing rapidly. It is anticipated that the use of LOD will have several advantages compared to current test methods. Prominent applications involve fast diagnostics at the location where diagnosis is needed (point of care) and often concern small amounts of sample and materials required to perform tests. However, care should be taken for quality management aspects regarding calibration and maintenance of the device, and training and education of the user is necessary. Centrifugal microfluidic platforms also require a minimal amount of instrumentation for fluid propulsion and eliminate the need for external syringe pumps. Centrifugal microfluidics is now mostly used in, e.g., disc-based PCR [19,20], quantifying original levels of genomic material [21], plasma separation [22,23], ELISA tests [24,25,26], colorimetric assays for the determination of alcohol or glucose concentration in whole blood [27,28] culturing roundworms [29,30], water, food and soil analysis [31,32,33], whole blood cells isolation [34,35] and in many other applications reviewed by, e.g., Gorkin et al. [36] and Strohmeier et al. [37].

## 2. Device Design and Operation

**Figure 1:**
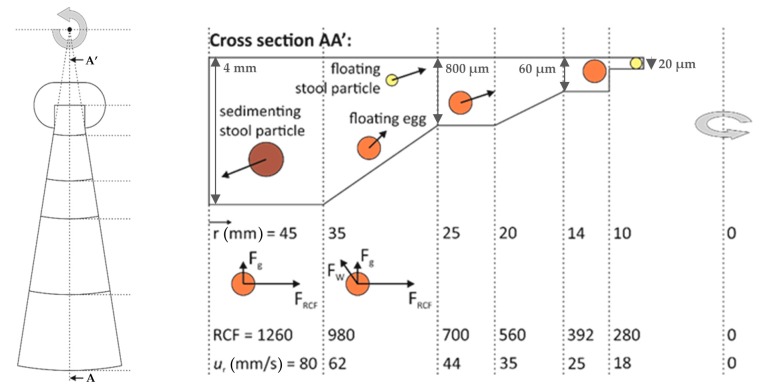
Working principle of the flotation chamber. Top and cross-sectional views illustrate the particle movement, where the acting forces on the eggs are drawn within their corresponding section. F_RCF_ is the relative centrifugal force caused by disc rotation, F_g_ is the natural buoyancy force (by Earth’s gravity), F_W_ is the wall reaction force acting on the particle against the chamber bottom surface. The drag force, which arises from the particle movement, which is equal in magnitude and opposite in direction to the illustrated total net forces, is not shown for simplicity. Although the top view is scaled correctly, the cross-sectional view is not on scale for clarity. The corresponding relative centrifugal force (F_RCF_) and radial velocity (net velocity towards the center of the disk) values are listed at the interfaces between segments, and the radial positions are also provided. The values are provided for a spin speed of 5000 rpm during 5 min (see calculations in Supplementary Figures S1 and S2).

Our purification device is based on the flotation behavior of the parasite eggs in flotation solutions like the above-mentioned state-of-the-art systems but we additionally implemented a number of new features that are essential for the successful and integrated functioning of the device (Figure 1). 

### 2.1. Guided Two-Dimensional (2D) Flotation

The first feature is the guided two-dimensional (2-D) flotation. This approach combines centrifugation enhanced and natural flotation behavior. The parasite eggs and the other floating particles within the stool sample injected into a prefilled chamber of the flat and rotating disk start their migration from the deepest and the widest section of the chamber (71% of the total volume) travelling towards the center of the disk by flotation, accelerated by the applied centrifugal force. Simultaneously, they float to the top of the chamber by the natural buoyancy force caused by the earth’s gravity in a perpendicular direction to their faster movement towards the center. This weaker force component is assisted by creating a secondary force on the same direction by guiding particles upward by applying an inclination with an ascending height to the bottom surface of the chamber, yielding a gradual decrease in chamber height while lifting the particles away from the wall [38]. 

### 2.2. Converging Chamber

The second feature is the converging chamber. From the circumference towards the center, the flow chamber decreases in both the height and the width to eventually make a transition to a shallow section with a rectangular shape. This collection chamber is designed such that it can host a packed monolayer of eggs for counting and identification. Three sides of this chamber are open and connected to a shallower section to allow smaller particles to continue floating towards the center, and to evacuate air bubbles from the collection chamber.

### 2.3. Continuous Size-Based Filtering

The third feature is the continuous size-based filtering. In practice, a band-pass filter, which is set for the parasite egg size, is applied to the floating particles. The ones larger than the eggs were blocked at different stages based on their size during the step-by-step application of the gradual decrease of the chamber depth prior to the collection zone, followed by the removal of the ones smaller than the eggs by further decreasing the height below the egg size. The last step also prevents the eggs proceeding further downstream (will be illustrated later).

### 2.4. Single-Shot Imaging

The fourth feature is the single-shot imaging. The parasite eggs are collected within the imaging zone (the collection chamber)—ideally as a packed monolayer allowing a single capture of a still image for subsequent processing for the manual or digital counting and identification of the eggs.

### 2.5. Design

The device is a flat rotating disk with 10 cm diameter, which can be replaced with the rotor of a commercial minicentrifuge. The disk consists of two identical flotation chambers, each having 1 mL of total volume and matching footprint when the disk is rotated 180°. This configuration enables not only parallel processing, but also to establish the counterbalance effect required for stable centrifugation. A hole of 6.3 mm diameter is placed at the center of the disk for positioning onto the attachment pin of the minicentrifuge. The imaging zone (collection chamber) is placed 10 mm further from the center towards the circumference (starts at the radial distance, r = 10 mm). The depth of 60–120 µm was set for forcing the formation of the monolayer of the eggs (size range: 60–100 µm) and the length and the width of this zone was defined as 2.5 mm × 4.0 mm to match the size of the imaging sensor. Around the imaging zone, a filtering unit, which has a depth of 20 µm and is connected to the outlet, is placed for the removal of the excessive content, i.e., particles smaller than 20 µm in diameter and air bubbles. One side of the imaging zone extends towards the circumference while radially expanding up to the radial distance of r = 14 mm.

**Figure 2:**
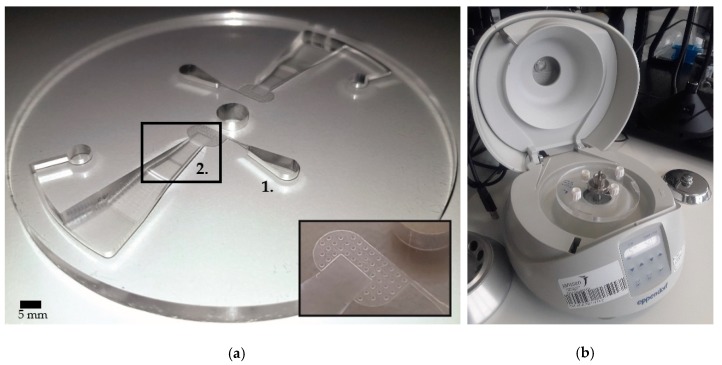
(**a**) Fabricated lab-on-a-disk device (without cover plate and before bonding) (**b**) and the commercial minicentrifuge with the device fixed onto its rotor. Inset in (**a**) shows a closer look into the collection/imaging zone and the downstream filtering unit with a depth of 20 µm equipped with pillars, which are 400 µm in diameter and have 400 µm spacing. The PMMA disk, hosting two identical chambers, has 10 cm in diameter and 5 mm in thickness. Chamber depth goes from 4 mm to 20 µm in steps. The center hole with a diameter of 6.3 mm is for fitting the disk onto the commercial minicentrifuge. (1.) shows the design of the outlet region, which is 2 mm in depth. (2.) shows a part of the flow chamber, where it converges to the collection/imaging zone with a depth going from 600 to 60 µm. The footprint of the imaging zone is measured 4 mm by 2.5 mm.

Following the arc at r = 45 mm, the injection arm with the width of 1 mm and the depth of 2 mm is extended from the chamber to make the connection to the outlet. Figure 2 shows the fabricated device and the minicentrifuge (Eppendorf, Germany) after the LOD device (with fluidic connections attached) was fixed. To avoid a random immobilization of the particles and blockage of the chamber, side walls of the converging chamber were designed to follow the lines towards the center of rotation (Figure 1). Since the vector that defines the direction of the created centrifugal force by the disk rotation is always towards the center of rotation, this design aspect avoids the possibility of particles crashing and sticking onto the side walls.

To avoid the formation of clusters, the chamber height was designed to be decreased in steps. This design aspect induces variations of the relative importance of different forces acting on the particles.

The pillars with 400 µm spacing in the area after the collection part were incorporated to avoid collapsing and completely blocking the 20 µm deep zone during bonding and manual pressing. 

## 3. Materials and Methods 

### 3.1. Fabrication

The devices were fabricated by computer numerical control (CNC) milling (Datron Neo, Datron) in polymethyl methacrylate (PMMA; Eriks). The total thickness of the stack was 7 mm, where the chamber and the cover with access holes were milled in plates of 5 mm and 2 mm, respectively. The CAD (computer-aided design, 2017) models were created in SolidWorks and transferred to SolidCAM for the automated milling operation. By defining all the parameters for each milling step using SolidCAM’s HSR (high speed roughing) and HSM (high speed machining) modules, the whole process was compiled into a single file and loaded into the CNC (computer numerical control) machine. The total milling time per device was around 45 min, followed by manual thread opening for the holes on the cover plate. After ultrasonic cleaning in 50:50 Isopropanol:DI (de-ionized water) solution for 10 min, the two PMMA disks were dried and placed on top of the (custom-designed) home-made aluminum bonding chambers, which were pre-filled with 3 mL of dichloromethane (DCM; Sigma-Aldrich) solution. Sealing the chambers with lids and the waiting time of 2.5 min allowed DCM to vaporize and condition the PMMA surface for bonding. After this treatment, the disks were immediately bonded together. No tools were required for bonding and manual pressing the stack was sufficient to achieve an appropriate pre-bond. The first few minutes are critical for manual inspection and removing the air bubbles trapped between the disks by applying gentle force by hand over the entire surface. Then, the stack was sandwiched between two plastic plates and placed under a weight of 10 kg for 30 min for the bonding to be completed. As a last step before the experiments, the plastic (Polypropylene) Luer-lock adapters (Cole-Parmer) were screwed into the inlets and the outlets. Luer-tip plastic syringes were used for infusing/discharging liquids into/from the device without leakage. The total weight of the device was around 50 g, which is less than the rotor of the minicentrifuge. The fabrication cost of the device is currently on the order of 15 €/disc, but it is estimated that this can be reduced well below 5 €/test.

### 3.2. Imaging Setup

**Figure 3:**
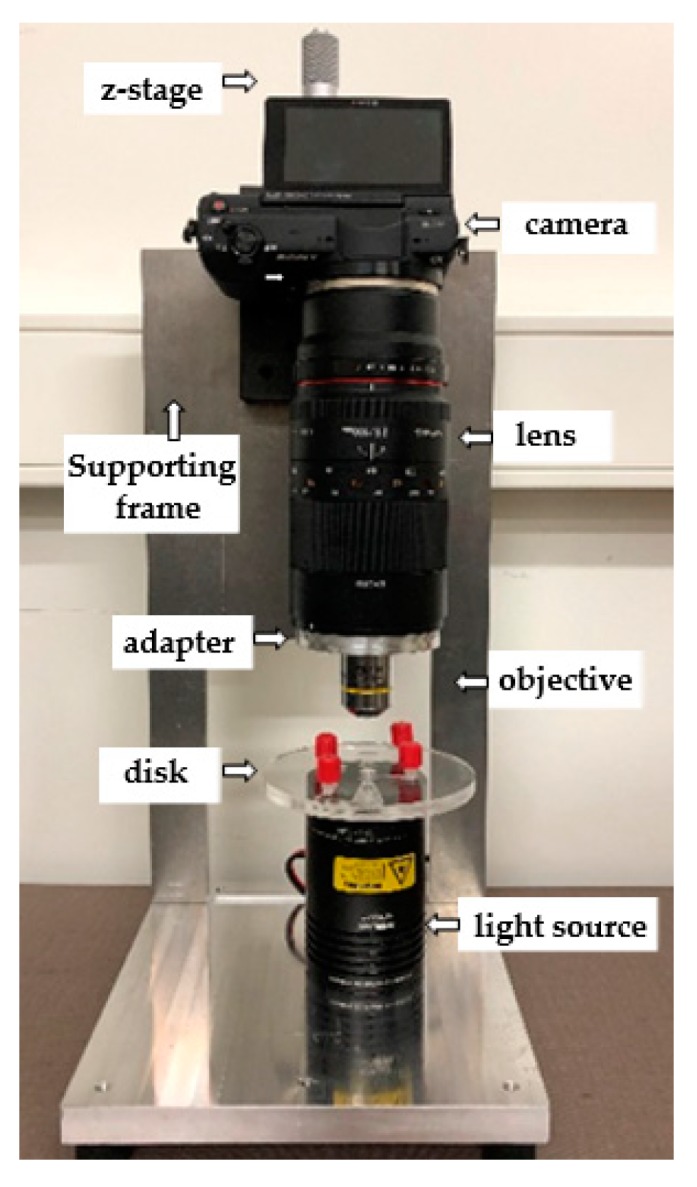
Picture of the imaging setup.

The imaging setup is designed to be a low-cost, bench-top system, which can be easily transferred for field experiments. To perform high-resolution single-shot imaging, a mid-level commercial digital camera, Sony α5100 (FotoKonijnenberg, NL), equipped with an APS-C digital CMOS image sensor with 24 MP (6000 px × 4000 px) resolution was selected. The camera was attached to a precise (10 µm accuracy, 25 mm range) single axis translation stage (Thorlabs), which was fixed onto a coarse (10 cm range) translation stage (Thorlabs), sitting on the main frame (Figure 3). A macrolens, Samyang 100 mm F2.8 (FotoKonijnenberg, NL) was attached to the camera, where an adapter was machined by CNC milling for fixing a 10× microscope objective (Olympus Plan Achromat, Thorlabs) onto the lens. For illumination, a halogen light source (Quartz Tungsten-Halogen Lamp, Thorlabs), equipped with a diffuser on top, was placed at the bottom, where the LOD device could directly be placed onto it, due to its large form factor. This allowed imaging in transmitted light mode, while illuminating the sample from its bottom face and imaging through its top face. The alignment of the imaging zone on the device to the field of view (FOV) of the imaging setup could easily be done by hand, therefore the setup was kept simple without an X–Y translation stage for the LOD device.

Using the direct Wi-Fi connection capability of the camera to any mobile device, an android tablet (Samsung Tab A) was connected to control the camera to take the images, and stored for inspection, analysis, and easy upload to the cloud. For the camera control, the free software from Sony (Play memories) on Google Play Store was used.

Since the magnification ratio of the objective was defined for typical optical configuration for microscopes (180 mm tube length), we calibrated our imaging setup (100 mm tube length) for determining its real magnification ratio. Measuring already known distances and calibrating the system, we found a 5.48× magnification. For the original sensor size of 24 mm × 16 mm and pixels of 4 µm, the field of view (FOV) is measured to be 4.38 mm × 2.92mm, resulting in 0.73 µm pixel size. Based on this calibration data, the imaging zone of the LOD device was defined as 2.5 mm × 4.5 mm. The total cost of the whole imaging setup was around 1200 € and of the centrifuge 1000 €.

To evaluate the performance of the LOD platform, infected pig and human samples were tested. The stool samples from infected pigs were donated by Prof. Bruno Levecke from Ghent University. The human stool samples were collected by Neglected Tropical Diseases lab of Prof. Zeleke Mekonnen at Jimma University (Ethiopia) in an endemic STH region. To allow testing of these fresh stool samples without preservation, the experiments with the human samples were performed at Jimma University (Ethiopia). 

## 4. Experimental

**Figure 4:**
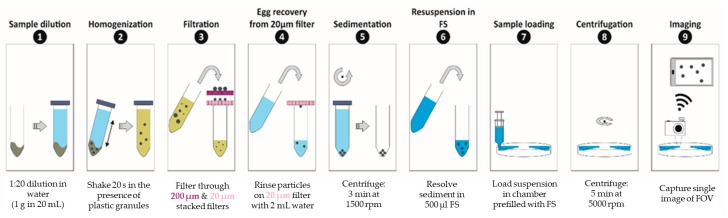
Layout description of the flow chamber and the illustrations of the steps of the centrifugation analysis.

The whole experimental procedure including the sample prep, centrifugation and imaging is summarized in Figure 4. For each experiment, the sample preparation procedure was performed in duplicate: one for testing in our LOD device and the other one for counting in a McMaster slide to get information on the amount of STH egg input in the LOD device. To evaluate the performance of the whole experimental procedure, the concentration of STH eggs in the stool samples were also examined with Mini-FLOTAC as a reference. In each technique eggs were counted manually.

### 4.1. Sample Preparation 

Fresh stool samples were obtained and stored at 4 °C until examination. Fecal sample of 1g was taken and diluted in 20 mL DI water in 50 mL Falcon tube (1:20 dilution ratio). For homogenization of the solution, plastic granules (Apacor) were added and the tube was shaken for about 20 s. Properly mixed sample was then filtered through stacked polyethylene terephthalate (PET) filters (pluriStrainer^®^) with 200 µm and 20 µm pore sizes to remove granules and particles bigger than 200 µm and smaller than 20 µm (see Supplementary Figures S3 and S4). Next, the remaining particles on a 20 µm filter surface were rinsed with 2 mL DI water to recover parasite eggs. After flushing, the rinsed solution was transferred to 2 mL centrifugation vial (Eppendorf) and centrifuged for 3 min at 1500 rpm (Eppendorf^®^ Minispin^®^). After centrifugation, the supernatant was removed and particles were resuspended in 500 µL flotation solution (FS) and transferred to a 3 mL syringe. Sample was always prepared in duplicate and divided into two syringes. One was injected into our LOD device (Supplementary Figure S5) and the other was used as a reference in the McMaster chamber. The flotation solution used for the experiments was FS2 (Sigma-Aldrich). This saturated sodium chloride solution with specific gravity of 1.2 and works best for Ascaris eggs (specific gravity of 1.11–1.20).

### 4.2. Centrifugation 

**Figure 5:**
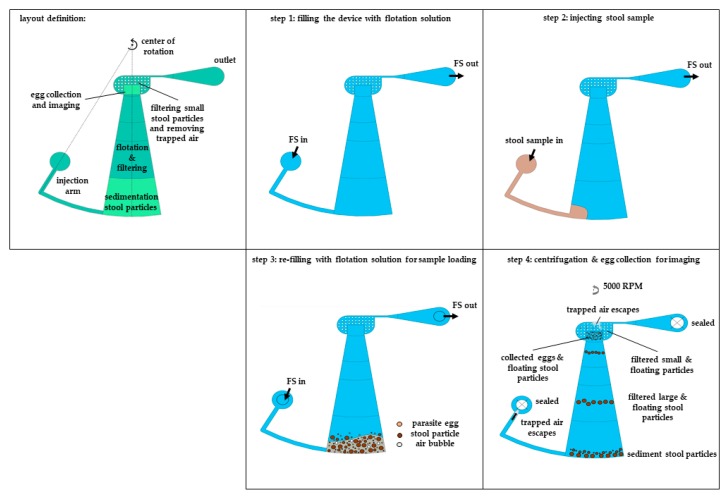
Layout description of the flow chamber and illustrations of the steps of the centrifugation analysis (illustration for section *2.3. Continuous Size-Based Filtering*).

As a first step, the chamber was filled with flotation solution without pre-treatment. Due to the design and placement of the injection arm and the converging layout of the chamber, the device pre-filling step was consistently successful without leaving any unwetted area in the chamber. As a second step, the stool sample was injected through the inlet, while the same amount of excessive flotation solution was extracted from the outlet. Infusion of the sample solution should be done slowly to avoid that particles prematurely reach the imaging zone. It is aimed to keep the sample within the first zone (4 mm deep section of the device). As a third step, the floatation solution was infused through the inlet until the remaining sample solution inside the injection arm was completely replaced by the flotation solution. Since the volume of the first zone is larger than 0.5 mL, sample volumes up to this value is possible with this device. After successful injection of the sample, inlet and outlet were sealed by Luer-lock caps and the LOD device was transferred and fixed onto the minicentrifuge, followed by centrifugation at 5000 rpm for 5 min. Finally, the device was taken out and the imaging zone was inspected, and the image was taken. Figure 5 gives an overview of the procedure (except the last imaging step).

To determine the optimal operational parameters, the spin speed and the duration were optimized by testing spin speeds starting from 2000 rpm and time periods between 1 and 10 min. It was observed that the spin speeds below 4000 rpm never yielded a successful collection of the eggs, regardless of the duration and extended durations at an optimal spin speed never resulted in an enhancement in separation, either (see Supplementary Figures S6–S8). 

## 5. Results and Discussion 

**Figure 6:**
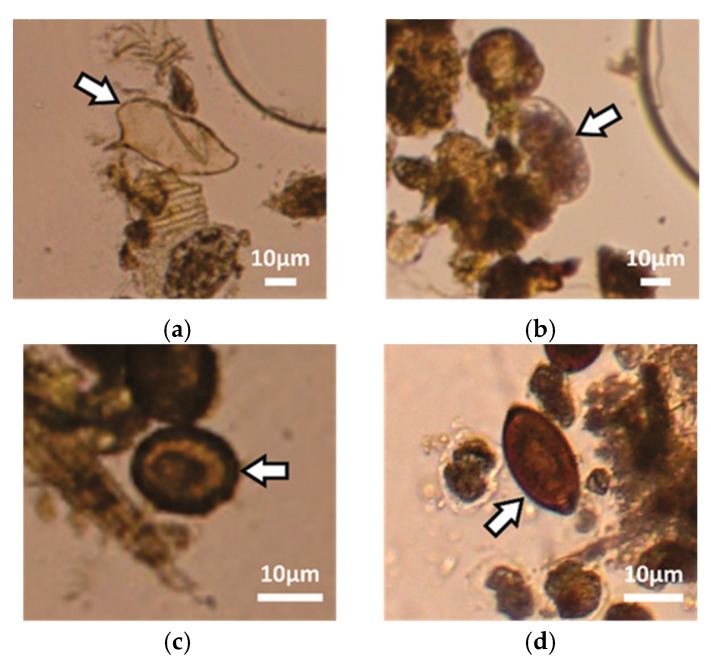
Digitally zoomed images of identified parasite eggs with our system (caption of Figure 2a): *Schistosoma mansoni* (**a**), hookworm (**b**), *Ascaris lumbricoides* (**c**) and *Trichuris trichiura* (**d**).

The sample set contained positive samples for the STH species (*Ascaris lumbricoides* and *Trichuris trichiura*), hookworm (*Necator americanus* and *Ancylostoma duodenale*) and *Schistosoma mansoni*. It was possible to capture eggs of any of these STH species and schistosomes with the LOD device and the image quality was appropriate for identification and counting of the eggs. Figure 6 shows digitally zoomed images of parasite eggs from the parasite species within the collection/imaging zone of the LOD device.

To assess the capability of the LOD device for the isolation of STH eggs present in the stool sample within the collection/imaging zone and the quality of the image created using the imaging setup for identification and counting, stool samples from STH infected pigs and humans (*n* = 7) were examined.

**Figure 7:**
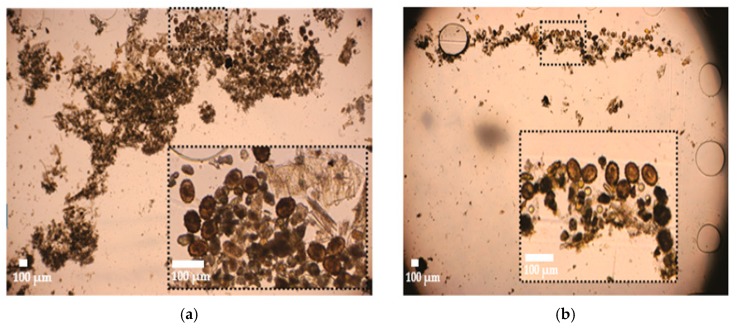
(**a**) Photo of the imaging zone (see Figure 2a) of the lab-on-a-disk device after testing a stool sample from a pig with *Ascaris suum* infection, and (**b**) photo of the imaging zone of the lab-on-a-disk device after testing a stool sample from human with *Ascaris* *lumbricoides* infection.

The results for the infected pig samples were compared with McMaster, the obtained recovery rates were minimally around 60% reaching up to 100% (Table 2). As described in the earlier sections, STH eggs are collected together with low density and small stool particles within the collection/imaging zone of the LOD device. Figure 7 shows pictures from this zone after centrifugation (a) of the pig stool sample infected with *Ascaris suum* and (b) of the infected human samples tested in Ethiopia. The depth of the collection zone was 60 µm, which forced formation of a monolayer of eggs, resulting in improved detection sensitivity and easier identification. 

The outcome of the field experiments for the infected human samples in Ethiopia was examined in more detail. To evaluate the isolation/capture efficiency of the LOD device, the whole chamber was inspected, and eggs were counted not only within the collection/imaging zone but also in the rest of the chamber. This showed that eggs indeed moved successfully towards the center of the LOD device with an average capture efficiency on the chip level of all samples (pig and human) of around 71% ± 26%. Of this amount 22% ± 16% was found in the field of view (see Table 2). Comparing the total egg counts in the LOD device immediately after injection to the total numbers obtained by counting the eggs present after the sample prep procedure (before injecting into the device) using McMaster slides suggested that a considerable amount of eggs was lost during injection (around 30% of eggs when using McMaster as reference, see Table 2). More eggs were inserted in the chip than detected in the original sample as obtained with reference method Mini-FLOTAC, which hints at considerable losses during quantification using Mini-FLOTAC. The loss during injection might be caused by sample transfer with a plastic syringe and the dead volume within the relatively large Luer-lock adapter. The capture efficiency can likely be further improved by avoiding adherence of the eggs to the substrate walls, by, e.g., applying a layer with an anti-stiction functionality. Reducing the syringe volume and using connections without dead volumes could also reduce egg loss. The results presented in the Table 2 also show that there was substantial loss of eggs inside the chip on the way to the field of view. Not all the eggs injected into the disk stay within the FOV. Almost half of the parasite eggs were detected in the zone after the FOV. A possible reason for eggs escaping from the FOV was that the examined human stool samples were mostly infected by hookworm parasites, which have more thin and flexible shells compared to *Ascaris* or *Trichuris* species. Thus, when the depth of the zone after the FOV was larger than 20 µm they could pass further. This problem can be solved by adjusting the current bonding procedure so that smaller pillar spacings can be attained.

**Figure 8:**
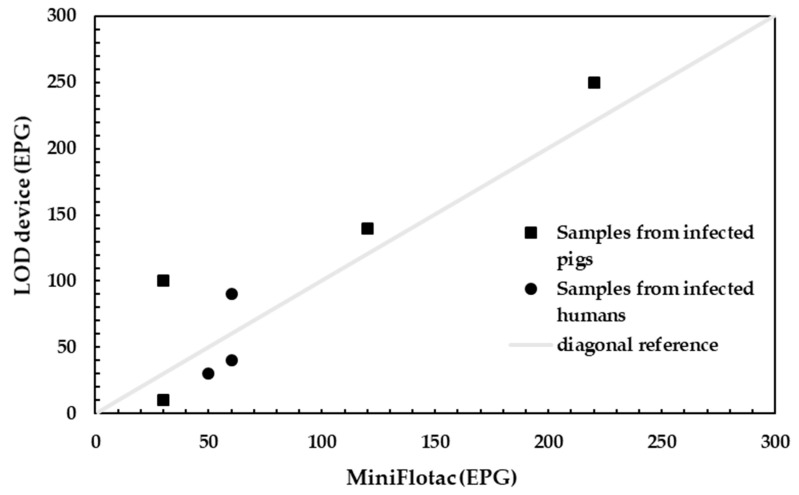
Amount of eggs present in soil-transmitted helminths (STH) infected samples detected with our device (y-axis) compared to Mini-FLOTAC (x-axis) for low infected samples.

STH eggs were successfully captured in the collection/imaging zone of the LOD device even for low infection levels below 250 EPG, where quantification was most relevant. Figure 8 shows the egg counts for this device versus Mini-FLOTAC. In terms of the counted number of eggs, the device correlated with Mini-FLOTAC with a correlation coefficient of 0.91. The samples with low egg counts in Mini-FLOTAC (between 30 EPG to 100 EPG) were still detectable with this device. 

## 6. Conclusions

We integrated egg flotation upon centrifugation into a lab-on-a-disk platform. Collection and identification of parasite eggs in stool was accomplished by utilizing a guided 2-D flotation with a converging chamber for continuous sized-based filtering and single-shot imaging. This device provided fast and efficient operation and an image of a packed monolayer of eggs collected within a single imaging zone. We added secondary floatation and size-based separation mechanisms for enabling the separation and to improve the purification efficiency. Having a monolayer of the eggs allows efficient light collection thereby ensuring optimal image quality. 

To provide a complete analysis platform, a low-cost, bench-top imaging setup to be coupled to the centrifugation unit, was designed and constructed. This platform was equipped with a high-resolution imaging unit, light source and ready for wireless data transfer. The entire platform was tested in Ethiopia on infected human samples for evaluation of the developed technology. The results successfully showed the potential of the concept. Further optimization of the device can be performed using computational fluid dynamics simulations.

Reaching accurate EPG counts with high recovery rates is important, however we also aimed to provide a high-quality single image allowing for precise egg identification and counting with this technology. Clear and sharp images of the eggs were obtained. The distinct advantage of this system is that a parasite egg monolayer can be formed by restricting the chamber height of the imaging zone to the size of a single egg (as low as 60 µm). The consumer camera that we have used can in future be replaced by a smartphone. This would allow for a further reduction of the instrument cost and can allow for a more widespread implementation of the technique.

Automation of the full sample preparation protocol was outside the scope of the present study and did not seem economic as filters would have to be included, which would be cumbersome and costly. The largest challenge is however the much larger volume used for the two filtration steps (respectively 20 and 2 mL) compared to the volume of the LOD channel circuitry (1 mL), which would, without dramatically changing the currently applied preparation approach, increase the footprint of the device dramatically. This would bring about additional fabrication costs but also considerable waste issues. Some steps can however be further automated in a straightforward manner. Loading of dispersed and diluted stool can be performed using a pump and a sample loop valve, which can be periodically cleaned after each injection.

Besides its essential aspects like fast operation and digitalization of the results, the technology has shown comparable performance to the existing systems.

## Figures and Tables

**Table 1:** Comparison of the major techniques for parasite detection in stool (info mainly from [11]).

Method	Principle	Advantage	Disadvantage	Sensitivity	Time	Sample Amount
Kato–Katz	Feces are pressed through a mesh screen to remove large particles. A portion of sieved sample is then transferred to the hole of a template on a slide. After filling the hole, the template is removed, and the remaining sample is covered with a piece of cellophane soaked in glycerol. The glycerol clears the fecal material from around the eggs.	Easy sample preparation Cheap	Reduced sensitivity in individuals with low parasite loads	Medium	30–60 min	41.7 mg
McMaster	Sample is added to a flotation solution and placed under a slide with two gridded chambers. Eggs float towards the surface and the ones within the gridded area of the chamber are counted using a microscope.	Easy procedure Fast results	Lacks sensitivity at low eggs counts	Medium	5–10 min	2 g
FECPAK	A tube with a central pillar is filled with a stool sample dissolved in flotation solution, allowing the parasite eggs to accumulate into a single viewing area within a fluid meniscus. An image of the fecal sample is then captured.	Digitalized images Doesn’t require technical skills	Limited sensitivity of the test	Medium	24 min	3 g
FLOTAC	Technique based on centrifugal flotation of a fecal sample suspension and subsequent translation of the apical portion of the floating suspension.	Very precise and sensitive	Complexity of the application Requirement for large swinging bucket centrifuge	Very high	12–15 min	1 g
Mini-FLOTAC	Method based on flotation of the eggs. Miniaturized version of FLOTAC. Two chambers (1 mL each) are filled with fecal sample diluted in flotation solution.	Permits work with fresh and fixed fecal samples No centrifugation steps	Detection of some parasites (e.g., trematoda) requires centrifugation	High	12 min	2 g
Cornell Wisconsin	Flotation of eggs in salt solutions is enabled by centrifugation. After centrifugation in a swinging bucket rotor, the eggs are collected on the top of the centrifugal tube meniscus, and subsequently transferred onto a cover slip and counted by light microscopy.	No expensive tools needed Cheap and easy procedure	Lack of precision, owing to the absence of a grid on the coverslip Low sensitivity	Low	20 min	5 g
Lab-on-a-disk	Separation is based on a combined gravitational and centrifugal flotation, with the eggs guided to a packed monolayer, enabling quantitation and identification of subtypes of the eggs present in a single field of view	Single and high quality digitalized image Fast results	Not commercially available Cost unclear	High	15 min	1 g

**Table 2:** Number of eggs per gram of stool (EPG) detected with the lab-on-a-disk device and compared to reference methods.

Sample	Reference Method * Total # of Eggs in the Sample (EPG)	Reference Method ** Total # of Eggs after Sample Preparation Steps (EPG)	# of Eggs in the FOV of Lab-on-a-Disk Device (EPG)	Total # of Eggs Inserted in the Lab-on-a-Disk Device (EPG)	Detection (%)		
					Loss Within the Chip	Total Eggs # Inserted in Chip vs. *	Total Eggs # Inserted in Chip vs. **
*Ascaris* infected pigs	120	250	70	140	50	116.67	56
*Ascaris* infected pigs	30	100	20	100	80	333.33	100
*Ascaris* infected pigs	30	10	0	10	100	33.33	100
*Trichuris* infected pigs	220	310	50	250	80	113.64	80.65
Human	50	115	5	30	83.33	60	26.09
Human	60	130	30	90	66.67	150	69.23
Human	50	65	5	40	87.50	66.67	61.54
Average ± standard deviation (%)					78 ± 16	125 ± 100	71 ± 26

* Mini-FLOTAC and ** McMaster.

## Supplementary Materials

The following are available online at https://www.mdpi.com/2072-666X/10/12/852/s1, Figure S1: Particle radial velocity chart, Figure S2: Relative centrifugal force chart, Figure S3: Results for sample with and without sample preparation including 20 μm filtering, Figure S4: Blocked disk after centrifugation without 20 μm filtering, Figure S5: Disk filled with stool sample before centrifugation, Figure S6: Pictures of the chip design without inclined bottom after centrifugation, Figure S7: Photos of the collection zone of the chip design with one inclined bottom step, Figure S8: Photos of the collection zone of the chip design with two inclined bottom steps. Taylor, J.R. *Classical Mechanics*; University Science Books: Sausalito, CA, USA, 2005. Stokes, G.G. On the effect of internal friction of fluids on the motion of pendulums. *Trans. Camb. Philos. Soc.* **1850**, *9*, 8–94.
