# Supplementary material for: Development of a Lab-on-a-Disk Platform with Digital Imaging for Identification and Counting of Parasite Eggs in Human and Animal Stool
Source: Micromachines (Basel). 2019 Dec 5;10(12):852. doi: 10.3390/mi10120852 (PMC6952989; doi:10.3390/mi10120852)
Supplement: Supplementary file 1 [file micromachines-10-00852-s001.pdf]

micromachines

MDPI

Supplementary materials: Development of a Lab-on-a-Disk Platform with Digital Imaging for Identification and Counting of Parasite Eggs in Human and Animal Stool

Sertan Sukas, Bieke Van Dorst, Agata Kryj, Ole Lagatie, Wim De Malsche and Lieven J. Stuyver

Supplementary Calculations

Calculation of centrifugal forces & corresponding migration velocities [39,40] (included in Figure 1):

Imported Mathcad file: Calculation of the forces acting on a spherical particle in the stationary fluid in a rotating disc:

particle radius $r_P = 25$ µm

particle volume, $V_P = 6.545 \times 10^{-14}$ m^3

$$V_p = \frac{4}{3} \cdot \pi \cdot {r_p}^3 \quad (1)$$

particle density $\rho_p = 1.11 \frac{g}{cm^3}$

fluid density $\rho_f = 1.2 \frac{g}{cm^3}$

angular velocity $\omega = 5000\ rpm$

radial position of the particle $r = 50\ mm,\ 49\ mm, 5\ mm$

Centrifugal force:

$$F_c(r) = \left(\rho_p - \rho_f\right) \cdot V_p \cdot \omega^2 \cdot r \quad (2)$$

fluid viscosity (ref: pubchem) $\mu = 1.93 \cdot 10^{-3}\ Pa \cdot s$

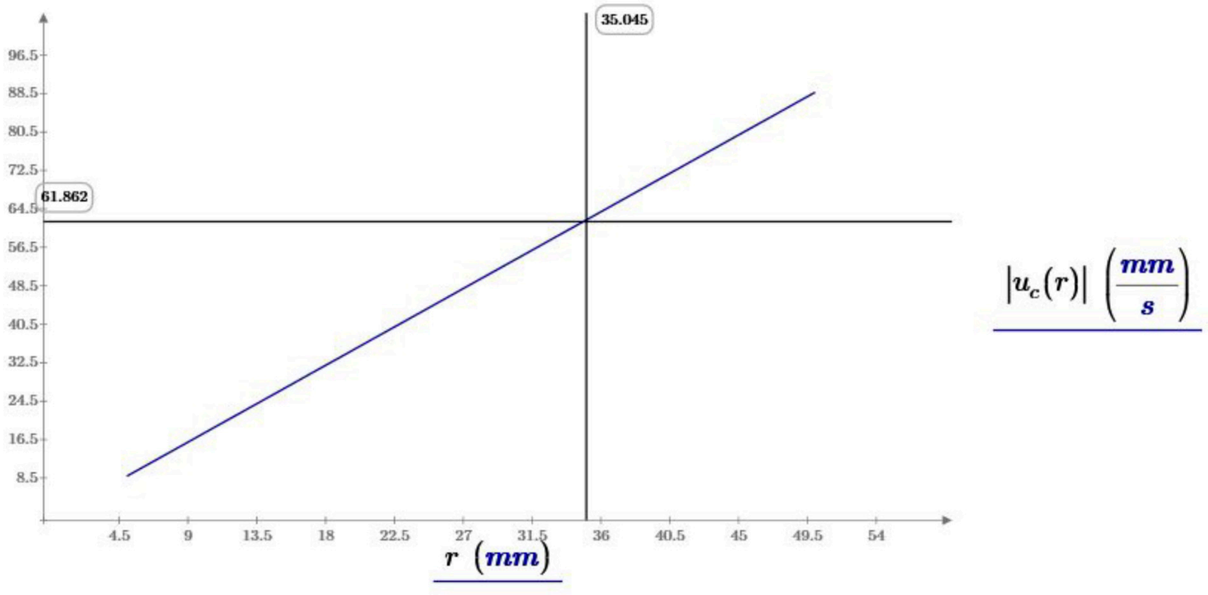

Figure S1. Particle radial velocity chart.

Particle radial velocity:

$$u_c(r) = \frac{F_c(r)}{6 \cdot \pi \cdot r_p \cdot \mu} \quad (3)$$

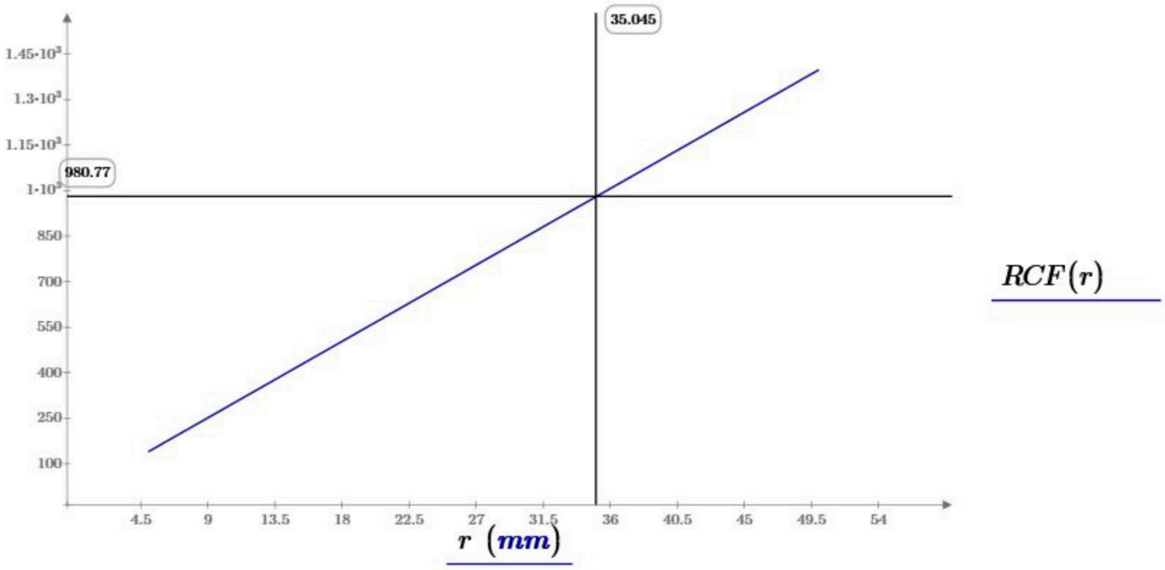

Figure S2. Relative centrifugal force chart.

Relative centrifugal force:

$$RCF(r) = \frac{r \cdot \omega^2}{g} \quad (4)$$

Supplementary Figures

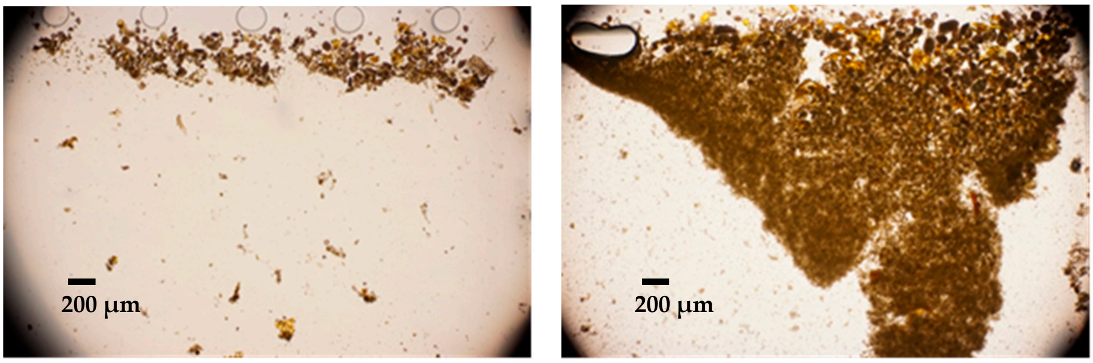

(**a**) (**b**)

Figure S3. Results for sample 401 (Table S1) with (left image) and without (right image) sample preparation including 20 µm filtering. This filtering step is essential for the developed sample preparation procedure within this study. On the right image, the entrance of the imaging/collection zone was already blocked by the small particles. Therefore, the centrifugation was inefficient, and recognition of the eggs captured in the collection zone became highly difficult.

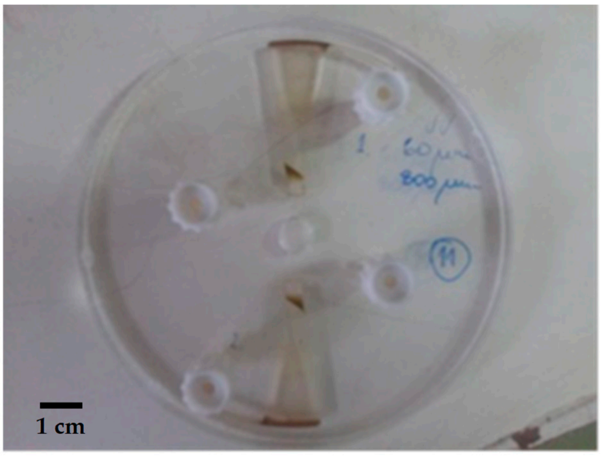

Figure S4. Blocked disk after centrifugation without 20 μm filtering (please refer to Figure S3).

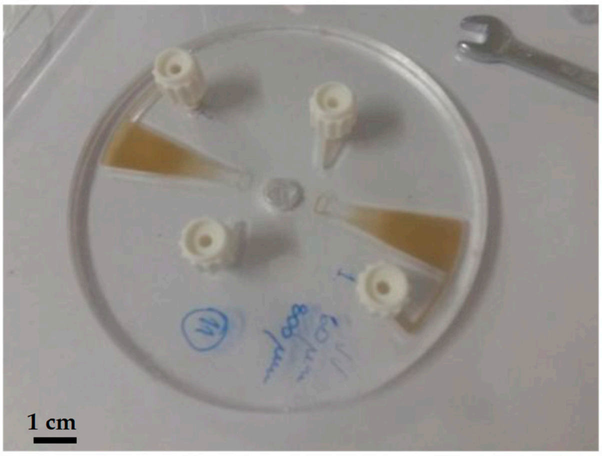

Figure S5. Disk filled with stool sample before centrifugation.

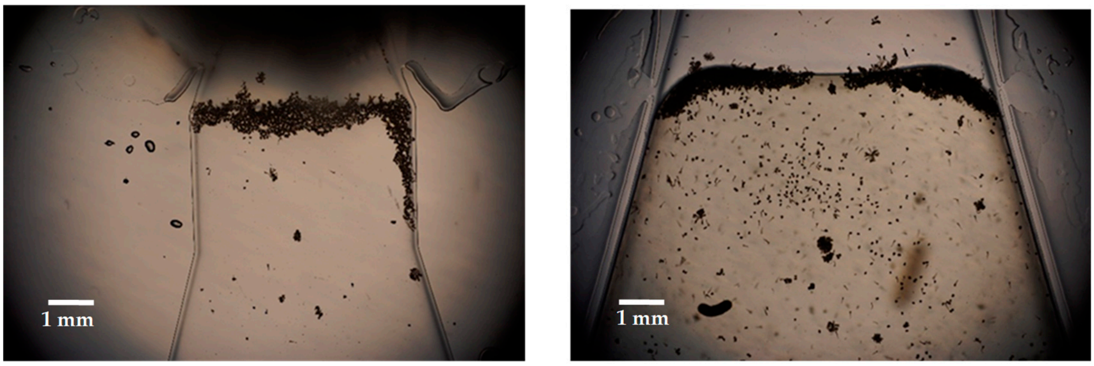

(**a**) (**b**)

Figure S6. Pictures of the chip design without inclined bottom after centrifugation: (**a**) photo of the collection area, (**b**) photo of the border between 4 mm deep chamber and 60 μm deep collection zone.

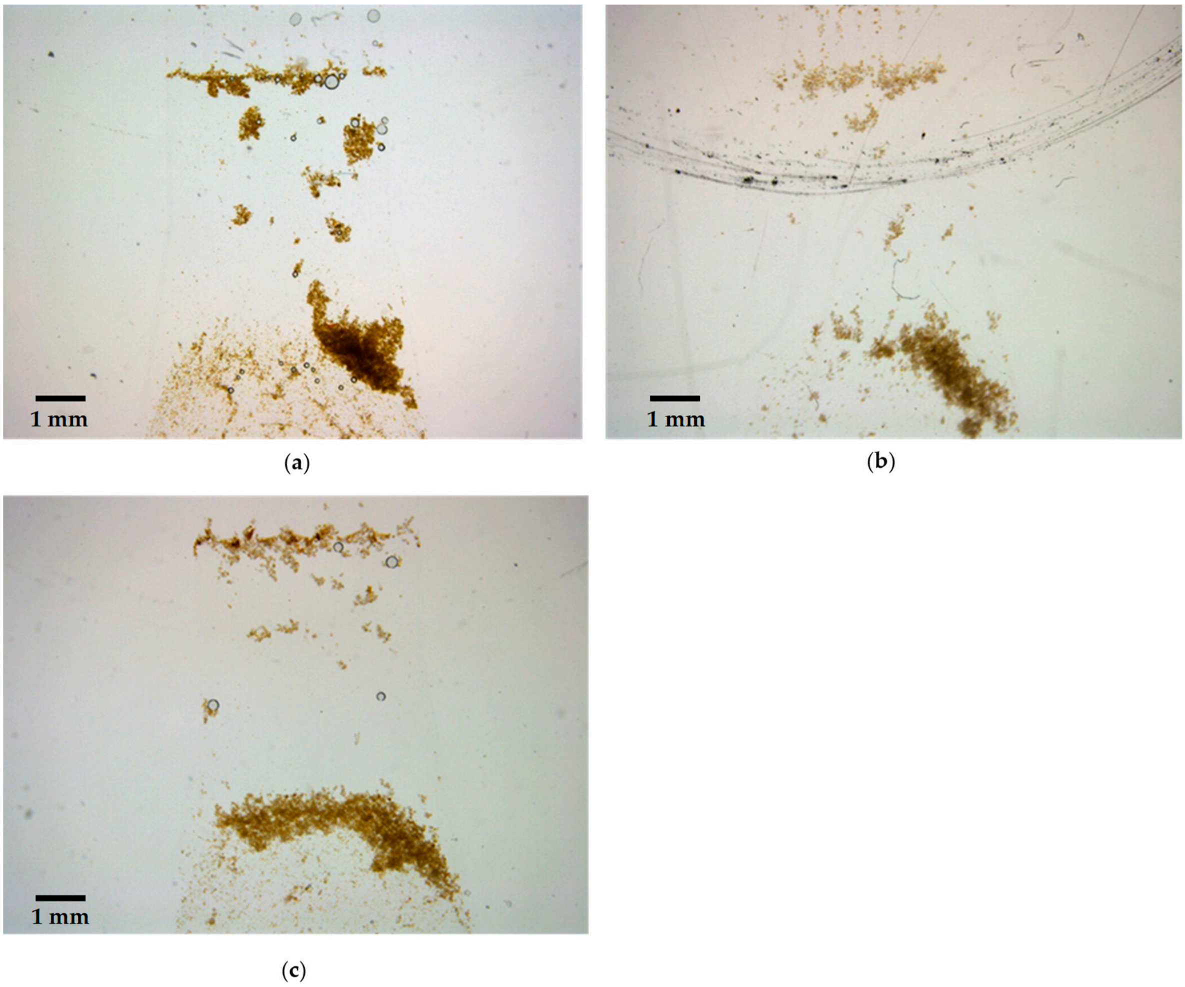

Figure S7. Photos of the collection zone of the chip design with one inclined bottom step. Photos of the chips with different samples after centrifugation at: **(a)** 2500 rpm for 3 min **(b)** 1500–3000 rpm for 2 min **(c)** 2000 rpm for 2 min.

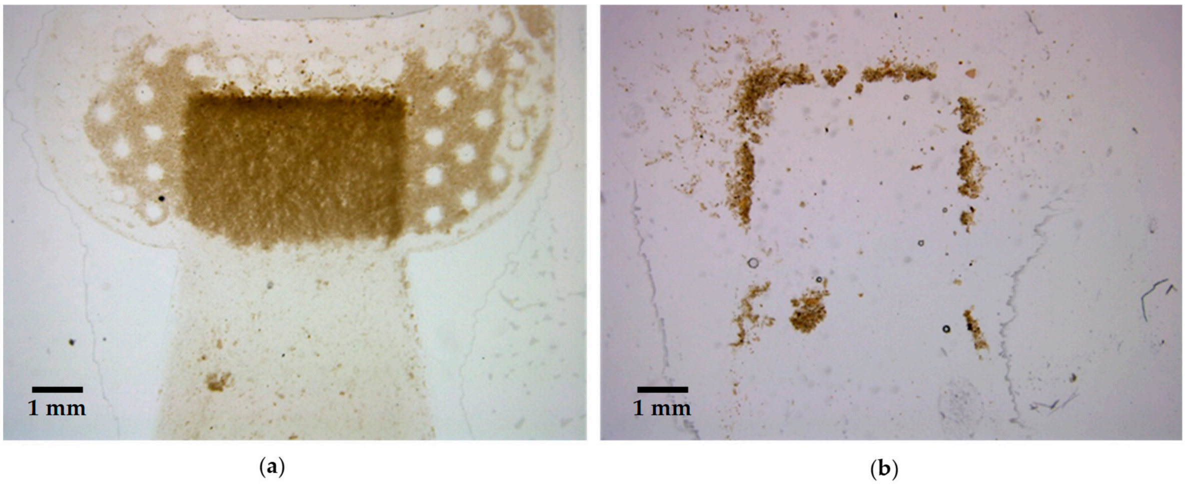

Figure S8. Photos of the collection zone of the chip design with two inclined bottom steps. Photos of the chip after centrifugation at 5000 rpm for 5 min: **(a)** sample with many small stool particles after Kato-Katz sample preparation procedure **(b)** sample after newly developed sample preparation procedure**.**
